# Einsatz von sedierenden Medikamenten und bewegungseinschränkenden Maßnahmen bei Patienten mit Demenz im Akutkrankenhaus: Eine nichtrandomisierte Fall-Kontroll-Studie

**DOI:** 10.1007/s00391-020-01697-3

**Published:** 2020-02-11

**Authors:** Daniel Lüdecke, Christopher Kofahl

**Affiliations:** Institut für Medizinische Soziologie, Universitätsklinikum Hamburg-Eppendorf, Martinistraße 52, 20246 Hamburg, Deutschland

**Keywords:** Innere Medizin, Versorgungsqualität, Versorgungskonzepte, Sedierung, Fixierung, Internal medicine, Quality of care, Special care concepts, Physical restraints, Chemical restraints

## Abstract

**Hintergrund:**

Auf Menschen mit Demenz mit stationär behandlungsbedürftiger Akuterkrankung ist der Großteil der Akutkrankenhäuser kaum vorbereitet. Dies birgt die Gefahr der Überforderung für das Personal. Demenzerkrankungen sind der häufigste Grund dafür, dass Krankenhauspersonal sedierende Medikamente verabreicht und bewegungseinschränkende Maßnahmen einsetzt.

**Zielsetzung:**

Die vorliegende Studie untersucht Faktoren, die den (unangemessenen) Einsatz von sedierenden Medikamenten und bewegungseinschränkenden Maßnahmen beeinflussen.

**Methoden:**

Eine nichtrandomisierte Fall-Kontroll-Studie wurde in 2 internistischen Abteilungen in Hamburg durchgeführt. In der Interventionsgruppe wurde ein spezielles Versorgungskonzept für Menschen mit Demenz implementiert. Die Versorgungsart in der Kontrollgruppe entsprach der Regelversorgung. Mit logistischen Regressionen wurden Zusammenhänge zwischen Faktoren wie Alter, Demenzschweregrad, Verhaltensauffälligkeiten, Barthel-Index oder Versorgungsart und dem Einsatz sedierender Medikamente bzw. bewegungseinschränkender Maßnahmen untersucht.

**Ergebnisse:**

Herausfordernde Verhaltensweisen (OR = 1,32) und die Zugehörigkeit zur Kontrollgruppe (OR = 1,94) sind signifikant mit dem Einsatz sedierender Medikamente assoziiert. Ein geringerer Barthel-Index, längere Aufenthaltsdauer und die eine Behandlung in der Kontrollgruppe sind signifikant mit einer höheren Wahrscheinlichkeit des Einsatzes bewegungseinschränkender Maßnahmen assoziiert.

**Diskussion:**

Der Einsatz sedierender Medikamente als auch bewegungseinschränkender Maßnahmen variiert stark zwischen Interventions- und Kontrollgruppe. Andere Studien, die zu ähnlichen Ergebnissen kommen, sehen verschiedene Bausteine spezieller Versorgungskonzepte als Gründe für diese Unterschiede. Dazu zählen neben der baulichen Gestaltung und räumlichen Aspekten auch demenzspezifische Schulungsangebote und ein angemessener Personalschlüssel. Dies vermag auch Unruhe und herausfordernde Verhaltensweisen aufseiten der Patienten zu reduzieren. Der Verzicht auf Sedierung und bewegungseinschränkende Maßnahmen hat nicht zuletzt auch positive Auswirkungen auf die Lebensqualität von Menschen mit Demenz.

## Abstract (en)

**Background:**

The majority of acute care hospitals are not prepared for people with dementia with acute diseases in need of treatment. This results in an increased likelihood of the personnel being overtaxed. Dementia is the most frequent reason that hospital personnel administer sedating medication and use restraining measures.

**Objective:**

The aim of this study was to investigate factors that influence the (inappropriate) use of sedating medication and physical restraints for patients with dementia in acute care hospitals.

**Methods:**

A non-randomized case control study, including two internal medicine wards was conducted in Hamburg, Germany. In the intervention group a special care concept was implemented focussing on patients with dementia, while the control group received regular care without a special dementia care concept. Logistic regression models were conducted to investigate associations between factors, such as age, severity of dementia, conspicuous behavior, Barthel index and type of treatment and the use of sedating medication and physical restraint measures.

**Results:**

Challenging behavior (odds ratio, OR = 1.32) and treatment in the control group (OR=1.94) were significantly associated with the use of sedating medication. A low Barthel index, longer periods of hospitalization and treatment in the control group were significantly associated with a higher probability of the implementation of physical restraining measures.

**Discussion:**

The use of sedating medication as well as physical restraining measures varied greatly between the intervention and control groups. This is in line with other studies that came to similar results and suggests that multiple components of special care concepts can explain these differences. These include architectonic design and spatial aspects as well as dementia-specific training of employees and an appropriate personnel complement. Such interventions can also reduce agitation and behavioral problems of patients. The avoidance of sedation and restraining measures is also positively associated with an increase in the quality of life of patients with dementia.

## Kurze Hinführung zum Thema

In Deutschland sind derzeit ca. 1,7 Mio. Menschen von einer Demenz betroffen. Menschen mit Demenz haben ein etwa gleich hohes akutmedizinisches Erkrankungsrisiko wie die übrige ältere Bevölkerung. Auf die über 60-Jährigen entfällt derzeit etwa die Hälfte aller Krankenhausaufenthalte. Schätzungen gehen davon aus, dass diese Zahl bis zum Jahr 2030 auf über 60 % steigen wird, wobei schon 2020 jeder 5. Krankenhausfall ein über 80-jähriger Patient sein wird. Deshalb ist von einer deutlichen Zunahme von Menschen mit Demenz im Krankenhaus auszugehen, worauf ein Großteil der Akutkrankenhäuser noch nicht ausreichend vorbereitet ist.

## Hintergrund und Fragestellung

Das durchschnittliche Alter von Patienten in der inneren Medizin ist in den letzten Jahren spürbar angestiegen und liegt mittlerweile bei 70 bis knapp unter 80 Jahren [10, 29]. Damit einhergehend nimmt auch der Anteil an Patienten mit demenziellen Erkrankungen (PmD) zu [18]. Die personelle Ausstattung und Qualifikation in Akutkrankenhäusern als auch die baulichen und räumlichen Bedingungen entsprechen häufig nicht hinreichend den besonderen Bedarfen und Bedürfnissen demenzerkrankter Patienten. Dies birgt die Gefahr der Verschlechterung des Gesundheitszustandes der Betroffenen sowie der Überforderung für das Personal [5, 24, 31].

In diesem Kontext steigt das Risiko erhöhter Behandlungskosten in der Akutversorgung durch Folgekomplikationen sowie durch erhöhte Rehabilitations- und/oder Pflegebedürftigkeit, z. B. durch weitere Demobilisierung infolge von bewegungseinschränkenden Maßnahmen und Fixierungen. So haben Demenzkranke ein doppelt bis dreifach erhöhtes Sturz- sowie ein bis zu vierfach erhöhtes Verletzungsrisiko [17, 23]. Insbesondere die Abteilungen der inneren bzw. internistischen Medizin sind überdurchschnittlich von Sturzproblemen betroffen [16]. Demenzerkrankungen sind der häufigste Grund dafür, dass Krankenhauspersonal bewegungseinschränkende Maßnahmen wie Seitenschutz/Bettseitenschutzleisten, Fixiergurte oder Therapietische einsetzt und sedierende Medikamente verabreicht [15, 22]. Der Einsatz dieser restriktiven Maßnahmen ist in erster Linie den Rahmenbedingungen der Akutkrankenhausabteilungen geschuldet, deren Arbeitsabläufe und räumlich-architektonische Bedingungen nicht auf die eingeschränkten kognitiven Fähigkeiten der demenzerkrankten Menschen ausgerichtet sind [13, 20, 21].

Um eine den besonderen Problemen der PmD angemessene Behandlung durchführen zu können, wurde im Evangelischen Krankenhaus Alsterdorf (EKA) in Hamburg ein neues Modellkonzept „Station DAVID“ (Diagnostik, Akuttherapie, Validation auf einer Internistischen Station für Menschen mit Demenz) implementiert, mit dem Ziel, durch besondere konzeptionelle Bausteine u. a. den Einsatz von bewegungseinschränkenden sowie Sedierungsmaßnahmen zu verringern und die Lebens- und Versorgungsqualität von PmD zu verbessern. In diesem Kontext sollen 2 Fragestellungen beantwortet werden: 1) Welche Faktoren beeinflussen den Einsatz von Sedierungen und bewegungseinschränkender Maßnahmen bei PmD? 2) Unterscheidet sich der Zusammenhang zwischen relevanten Einflussfaktoren und dem Einsatz von bewegungseinschränkenden und Sedierungsmaßnahmen in Abhängigkeit davon, ob sich die Versorgung an einem speziellen Konzept für Demenzpatienten orientiert oder es sich um eine Regelversorgung handelt?

## Studiendesign und Untersuchungsmethoden

### Studiendesign

**Figure 1:**
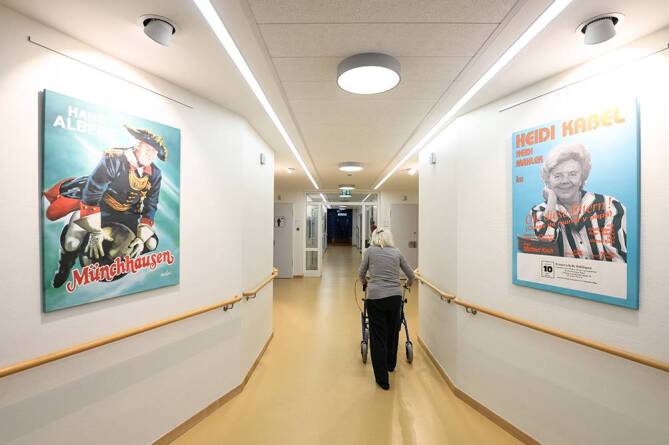

**Figure 2:**
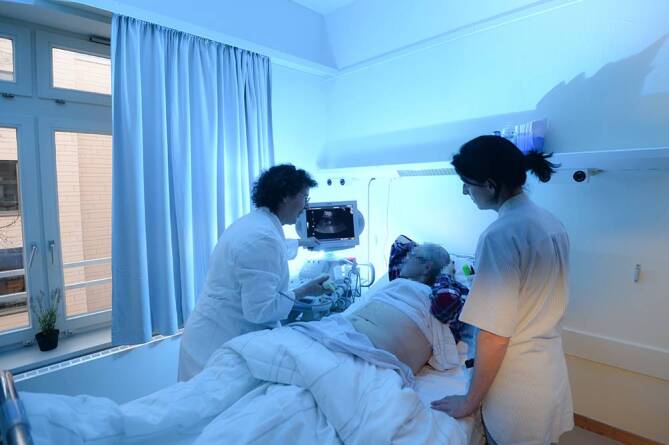

Das Ziel der vorliegenden Studie war der Vergleich eines speziellen Versorgungskonzepts für PmD (Interventionsgruppe) mit der Regelversorgung ohne entsprechendes Spezialkonzept (Kontrollgruppe) in Akutkrankenhäusern. Die Studie wurde als nichtrandomisierte Fall-Kontroll-Studie konzipiert, an der 2 Abteilungen der inneren Medizin aus verschiedenen Krankenhäusern in Hamburg teilnahmen. In der Interventionsgruppe wurde ein Versorgungskonzept implementiert^1^, das sich an den besonderen Bedürfnissen von PmD orientierte. Dies umfasste mehrere Bausteine, wie beispielsweise a), da die Station neu gebaut wurde, konnten besondere bauliche und farbliche Gestaltungen von Fluren, Türen oder Zimmern berücksichtigt werden (Abb. 1), wie z. B. mindestens 500 lx Lichtstärke auf Augenhöhe oder wohnzimmerähnlicher Aufenthaltsraum für die Tagesbetreuung; b) umfassende Schulungen sowohl des medizinischen und ärztlichen Personals, aber auch des Servicepersonals im Umgang mit Demenzerkrankten, u. a. in Validation, Biografiearbeit oder basaler Stimulation; darüber hinaus gab es regelmäßige Fallkonferenzen, in denen besprochen wurde, wie herausfordernden Verhaltensweisen am besten begegnet werden kann; c) mobile Diagnostikgeräte, um Ortswechsel und damit einhergehende Unruhe und Orientierungsschwierigkeiten zu vermeiden (Abb. 2); d) Einbindung von Angehörigen in die Versorgungs- und Entlassungsplanung sowie „Rooming-in“-Angebote (Angehörigen wurde die Möglichkeit eröffnet, zusammen mit den Patienten in einem Zimmer zu übernachten, sofern nicht bereits weitere Patienten in diesem Zimmer lagen); e) regelmäßige therapeutische Angebote. Um das Konzept adäquat umzusetzen, gab es einen höheren als auf internistischen Abteilungen üblichen Personalschlüssel. Die Versorgung in der Kontrollgruppe entspricht der Regelversorgung ohne besondere Bausteine der Dementenversorgung. Systematische Schulungsangebote für das Personal im Umgang mit Demenzerkrankten gab es nicht. Die baulichen Gegebenheiten entsprachen den üblichen Standards in der Regelversorgung in der inneren Medizin und umfassten keine speziell auf Demenzerkrankte abgestimmten Elemente.

### Rekrutierung

Zu Beginn der Studie wurde ein Assessment-Fragebogen entwickelt, um die Daten von PmD zu erfassen. In beiden Krankenhäusern wurden „study nurses“ geschult, die die Patienten-Assessments im Zeitraum Juli 2015 bis September 2016 durchführten. In der Interventionsgruppe wurden 2 Study nurses eingesetzt (zu je 25 % einer Vollzeitstelle), in der Kontrollgruppe eine (zu 50 % einer Vollzeitstelle). Patienten wurden in die Studie eingeschlossen, wenn sie mindestens leichte kognitive Beeinträchtigungen aufwiesen oder eine Demenzdiagnose vorhanden war. Ausschlusskriterien waren das Fehlen demenzieller Symptome, vollständige Bettlägerigkeit bzw. Immobilität sowie eine Aufenthaltsdauer von mehr als 4 Wochen (um geriatrische Fälle von üblichen internistischen Behandlungsfällen abzugrenzen). Die Gesamtstichprobe betrug *n* = 521 PmD (Interventionsgruppe: *n* = 330; Kontrollgruppe: *n* = 191).

### Instrumente

#### Abhängige Variablen.

Sedierung war definiert als Gabe von Medikamenten mit ruhigstellender Wirkung, die im Krankenhaus als Bedarfsmedikation angesetzt und entsprechend verabreicht wurde (ja/nein). Dies umfasste Substanzen wie Neuroleptika, Benzodiazepine, Antidepressiva oder sonstige Antipsychotika und Sedativa. Eine bereits vor dem Krankenhausaufenthalt bestehende Regelmedikation von potenziell ruhigstellenden Medikamenten wurde zwar ebenfalls erhoben, bei der Analyse jedoch nicht berücksichtigt, um nur den akuten Einsatz dieser Medikamentengruppe zur Sedierung von Patienten bei herausfordernden Verhaltensweisen zu erfassen. Der Einsatz bewegungseinschränkender Maßnahmen umfasste Maßnahmen wie das Anbringen von Bettgittern oder Bettseitenstützen, Therapietische oder Tischbretter, die das Aufstehen verhinderten, und Fixiergurte. Hier wurde unterschieden, ob diese gar nicht oder mehrmals bis regelmäßig zum Einsatz kamen.

#### Unabhängige Variablen.

Erfasst wurden die Dauer des Aufenthalts in Tagen sowie Alter und Geschlecht der Patienten. Der Demenzschweregrad wurde mithilfe der Minimental State Examination (MMSE, [7]) auf einer Skala von 0 (sehr starke kognitive Einschränkungen) bis 30 (gar keine kognitiven Einschränkungen) gemessen und, basierend auf der ICD-10-Klassifikation, in 3 Schweregrade eingeteilt: schwere Demenz (0 bis 16 Punkte), mittelschwere Demenz (17 bis 23 Punkte) und leichte Demenz (24 bis 27 Punkte) [4]. Neben der Hauptdiagnose wurden im Assessment bis zu 5 weitere Nebendiagnosen abgefragt. Daraus wurde der Charlson-Komorbiditätsindex (CCI, [3]) erstellt, um die Morbidität der Patienten zu erfassen. Nach einem Beobachtungszeitraum von etwa einer Woche wurde mithilfe der Pittsburgh Agitation Scale (PAS) die Agitation bei den Patienten erfasst [27]. Die Skala reichte von 0 bis 16 Punkten, wobei ein höherer Wert bedeutet, dass stärkere Verhaltensauffälligkeiten vorliegen. Eine dichotome Variable „Versorgungsart“ mit den Ausprägungen „Interventionsgruppe“ vs. „Kontrollgruppe“ wurde als Indikator für die Interventions- bzw. Kontrollgruppe in die Analysen aufgenommen. Zudem wurden Sturzereignisse erfasst (mindestens ein vs. kein Sturz während des gesamten Aufenthalts).

### Statistische Auswertung

Die Beschreibung der Stichprobe erfolgte anhand deskriptiver Statistiken; signifikante Unterschiede wurden in Abhängigkeit der Skalenniveaus und Verteilungen der Variablen mittels t‑Tests, Mann-Whitney-U-Tests oder Chi-Quadrat-Tests bestimmt. Insgesamt wurden 4 logistische Regressionsmodelle berechnet. Modell 1 analysierte die Zusammenhänge zwischen Prädiktoren und Sedierung, während Modell 2 Prädiktoren untersuchte, die im Zusammenhang mit dem Einsatz bewegungseinschränkender Maßnahmen standen. „Odds ratios“ wurden als Effektstärken berichtet. Post hoc wurden die Modelle 1 und 2 um Interaktionsterme zwischen signifikanten Prädiktoren und der Versorgungsart ergänzt, und es wurde geprüft, ob die Zusammenhänge zwischen signifikanten Prädiktoren und den abhängigen Variablen nach Versorgungsart variierten. Sämtliche kontinuierliche Variablen (Aufenthaltsdauer, Patientenalter, CCI, Barthel-Index und PAS) wurden standardisiert. Alle Analysen wurden mit dem R Statistikpaket (Wien, Österreich) durchgeführt [25].

## Ergebnisse

### Beschreibung der Stichprobe

Die Verteilung der Patientencharakteristika, insgesamt und nach Krankenhäusern differenziert, beschreibt Tab. 1. Der Anteil an weiblichen Patienten der Stichprobe war 60,5 %. Dieser unterschied sich nicht signifikant zwischen den beiden Krankenhausabteilungen. Das durchschnittliche Alter lag bei 80,5 Jahren, wobei der Altersdurchschnitt in der Kontrollgruppe 4,1 Jahre höher war. Die Verteilung der Demenzschweregrade ist in beiden Gruppen ähnlich. Knapp zwei Drittel der Patienten hatten eine schwere Demenz, gut ein Viertel eine Demenz mittleren Schweregrads, und etwas unter 10 % der Patienten wiesen eine leichte Demenz auf. In der Interventionsgruppe war der CCI mit 2,5 Punkten geringfügig, dennoch signifikant geringer als in der Kontrollgruppe (3,2 Punkte). Demzufolge waren Patienten in der Kontrollgruppe mit einem Barthel-Index von durchschnittlich 30,0 Punkten auch signifikant schlechter bezogen auf ihre Alltagskompetenzen als Patienten in der Interventionsgruppe (40,9 Punkte). Die durchschnittliche Verweildauer lag insgesamt bei 9,1 Tagen und war in der Interventionsgruppe mit 9,5 Tagen um etwa einen Tag höher als in der Kontrollgruppe (8,4 Tage). Die Patienten in der Interventionsgruppe waren etwas weniger verhaltensauffällig als in der Kontrollgruppe (3,0 zu 3,9 Punkten). In der Kontrollgruppe wurden sedierende Medikamente bei 25,7 % aller Patienten gegeben, und bei 54,5 % der Patienten wurden bewegungseinschränkende Maßnahmen vorgenommen (gegenüber 14,2 und 27,9 % in der Interventionsgruppe).

**Table 1:** 

Merkmal	Interventionsgruppe (*n* = 330)	Kontrollgruppe (*n* = 191)	Gesamt (*n* = 521)	*p*-Werte für den Unterschied
Weiblich, %	61,2	59,2	60,5	0,713
Alter (SD)	79,0 ( ± 11,9)	83,1 ( ± 7,2)	80,5 ( ± 10,6)	*<0,001*
Leichte Demenz, %^a^	9,4	7,9	8,8	0,662
Mittlere Demenz, %^a^	27,0	29,8	28,0	0,547
Schwere Demenz, %^a^	63,6	62,3	63,1	0,834
Charlson-Komorbiditätsindex (SD)^b^	2,5 (± 2,0)	3,2 (± 3,0)	2,8 (± 1,6)	*<0,001*
Barthel-Index (SD)^c^	40,9 (± 30,4)	30,0 (± 28,0)	36,9 (± 30,0)	*<0,001*
Aufenthaltsdauer in Tagen (SD)	9,5 (± 5,0)	8,4 (± 4,9)	9,1 (± 5,0)	*0,002*
PAS-Gesamtscore (SD)^d^	3,0 (± 3,2)	3,9 (± 3,1)	3,3 (± 3,2)	*<0,001*
Einsatz bewegungseinschränkender Maßnahmen (ja), %	27,9	54,5	37,6	*<0,001*
Sedierung (ja, bei Bedarf), %	14,2	25,7	18,4	*0,002*
Sturzereignis, %	9,1	10,5	9,6	0,718

^a^ *Demenz (MMSE)*: leicht: 24–27; mittel: 17–23; schwer: ≤16

^b^ *Charlson-Komorbiditätsindex*: 0–9 (höher: stärkere Morbiditätslast)

^c^ *Barthel-Index*: 0‑100 (höher: bessere Mobilität)

^d^ *PAS (Pittsburgh Agitation Scale)*: 0–16 (höher: stärkere Aggressivität und Unruhe)

### Ergebnisse der Regressionsanalysen

Die Ergebnisse der Regressionsmodelle 1 und 2 zeigt Tab. 2. Bezogen auf Modell 1 lässt sich konstatieren, dass Patientenalter und -geschlecht, CCI, Barthel-Index sowie Aufenthaltsdauer nur sehr schwache Zusammenhänge mit einer Sedierung aufwiesen; zudem ist keiner dieser Prädiktoren signifikant. Ein höherer Demenzschweregrad weist zwar eine deutlich verringerte Wahrscheinlichkeit für Sedierungsmaßnahmen auf (OR 0,51), jedoch waren die Daten kompatibel mit möglichen OR von 0,21 bis 1,23 (95 %-KI), womit auch für das Merkmal „Demenz“ kein statistisch signifikanter Einfluss festgestellt werden konnte. Herausfordernde Verhaltensweisen (PAS, OR = 1,32) und Versorgungsart (OR = 1,94) hingegen hängen signifikant mit dem Einsatz sedierender Medikamente zusammen. Im Modell 2 waren ein geringerer Barthel-Index, längere Aufenthaltsdauer und die Versorgung in der Kontrollgruppe signifikant mit einer höheren Wahrscheinlichkeit assoziiert, dass bewegungseinschränkende Maßnahmen durchgeführt wurden.

**Table 2:** 

	Modell 1: Einflussfaktoren auf den Einsatz sedierender Medikamente			Modell 2: Einflussfaktoren auf den Einsatz bewegungseinschränkender Maßnahmen		
*Prädiktor*	*OR*	*95* *%-KI*	*p*	*OR*	*95* *%-KI*	*p*
Patientengeschlecht (Referenz: männlich)	1,05	0,66–1,68	0,839	0,77	0,49–1,22	0,267
Alter des Patienten	0,92	0,72–1,19	0,543	0,81	0,63–1,04	0,100
*Demenzschweregrad (Referenz: leicht)*						
Demenzschweregrad: mittel	0,83	0,36–1,93	0,668	0,66	0,23–1,91	0,446
Demenzschweregrad: schwer	0,51	0,21–1,23	0,136	0,95	0,34–2,67	0,918
Charlson-Komorbiditätsindex	0,99	0,78–1,26	0,965	1,02	0,80–1,30	0,856
Barthel-Index	0,89	0,67–1,17	0,394	0,22	0,16–0,31	*<0,001*
Aufenthaltsdauer (in Tagen)	1,02	0,81–1,28	0,873	1,37	1,09–1,71	*0,006*
PAS-Gesamtscore	1,32	1,06–1,66	*0,014*	1,28	1,00–1,63	0,051
Versorgungsart (Referenz: Interventionsgruppe)	1,94	1,20–3,15	*0,007*	3,12	1,92–5,07	*<0,001*

*Modell 1*: Haupteffekte, abhängige Variable „Sedierung“

*Modell 2*: Haupteffekte, abhängige Variable „Einsatz bewegungseinschränkender Maßnahmen“

Die Ergebnisse der Modelle 3 und 4 beschreibt Tab. 3. In diesen Modellen wurde zusätzlich ein Interaktionsterm der signifikanten Prädiktoren aus den Modellen 1 und 2 und der Variable „Versorgungsart“ gebildet. Die Ergebnisse aus den Modellen 1 und 2 spiegelten sich weitestgehend auch in den Modellen 3 und 4 wider. Darüber hinaus zeigte sich, dass sich die Zusammenhänge zwischen den Prädiktoren Barthel-Index, Aufenthaltsdauer sowie PAS und den jeweiligen abhängigen Variablen nicht statistisch signifikant nach Versorgungsart unterschieden.

**Table 3:** 

	Modell 3: Einflussfaktoren auf den Einsatz sedierender Medikamenten			Modell 4: Einflussfaktoren auf den Einsatz bewegungseinschränkender Maßnahmen		
	*OR*	*95* *%-KI*	*p*	*OR*	*95* *%-KI*	*p*
Patientengeschlecht (Referenz: männlich)	1,03	0,64–1,65	0,902	0,75	0,47–1,20	0,233
Alter des Patienten	0,93	0,72–1,19	0,561	0,82	0,64–1,05	0,114
Demenzschweregrad: mittel	0,83	0,36–1,94	0,670	0,64	0,23–1,93	0,405
Demenzschweregrad: schwer	0,52	0,22–1,26	0,149	0,93	0,34–2,75	0,890
Charlson-Komorbiditätsindex	1,00	0,78–1,26	0,970	1,02	0,80–1,30	0,867
Barthel-Index	0,88	0,67–1,16	0,376	0,25	0,17–0,37	*<0,001*
Aufenthaltsdauer (in Tagen)	1,02	0,81–1,28	0,880	1,39	1,06–1,84	*0,020*
PAS-Gesamtscore	1,41	1,06–1,87	*0,017*	1,26	0,99–1,62	0,059
Versorgungsart (Referenz: Interventionsgruppe)	1,99	1,22–3,24	*0,006*	2,74	1,60–4,70	*<0,001*
Versorgungsart*PAS	0,86	0,56–1,32	0,487	–	–	–
Versorgungsart*Barthel-Index	–	–	–	0,69	0,36–1,29	0,252
Versorgungsart*Aufenthaltsdauer	–	–	–	0,98	0,61–1,58	0,924

*Modell 3*: Haupteffekte und Interaktion (Versorgungsart*PAS), abhängige Variable „Sedierung“

*Modell 4*: Haupteffekte und Interaktion (Versorgungsart*PAS, Versorgungsart*Aufenthaltsdauer; Versorgungsart*Barthel-Index), abhängige Variable „Einsatz bewegungseinschränkender Maßnahmen“

## Diskussion

In dieser Studie wurde untersucht, welche Faktoren mit dem Einsatz sedierender Medikamente und bewegungseinschränkender Maßnahmen bei PmD in internistischen Abteilungen zusammenhingen, und ob sich diese Zusammenhänge zusätzlich in Abhängigkeit der Versorgungsart (Interventions- vs. Kontrollgruppe) unterschieden.

Im Modell 1 waren von allen patientenbezogenen Merkmalen die Verhaltensauffälligkeiten der einzige statistisch signifikante Prädiktor. Dieses Resultat entspricht nur z. T. den Ergebnissen aus anderen Studien, wo zusätzlich kognitive Beeinträchtigungen bzw. der Demenzschweregrad mit einer erhöhten Sedierung assoziiert war [15, 28]. Um zu prüfen, inwieweit inhaltliche Überschneidungen der Prädiktoren Demenz und Verhaltensauffälligkeiten vorliegen, wurden die Modelle auf Multikollinearität geprüft. Jedoch konnte keine auffällige Korrelation zwischen den Prädiktoren festgestellt werden. Dennoch kann davon ausgegangen werden, dass bei Patienten mit schwerer Demenz insbesondere die bei dieser Erkrankung häufig auftretenden herausfordernden Verhaltensweisen ausschlaggebend für vermehrte Sedierung sind. Studien zeigten zudem, dass der missbräuchliche Einsatz dieser Medikamente – vermutlich aufgrund ihrer sedierenden Wirkung – zu einem erhöhtem Sturzrisiko führt [26]. Zumindest in unseren Daten war Sedierung nicht signifikant mit Sturzereignissen assoziiert.

Von den patientenbezogenen Merkmalen in Modell 2 war der Barthel-Index ein signifikanter Prädiktor. Ferner stiegt mit zunehmender Aufenthaltsdauer die Wahrscheinlichkeit, dass bewegungseinschränkende Maßnahmen bei Patienten angewendet wurden. Ähnliche Ergebnisse zeigten sich auch in anderen Studien, in denen funktionelle Einschränkungen (Barthel-Index) die Wahrscheinlichkeit des Einsatzes bewegungseinschränkender Maßnahmen erhöhen [9, 21]. Dies lässt vermuten, dass bei Patienten mit eingeschränkter Mobilität die Sorge einer erhöhten Sturzgefahr vorliegt. Dies kann durch unsere Daten nicht bestätigt werden. In beiden Krankenhäusern gab es einen vergleichbaren Anteil an Sturzereignissen bei PmD, obgleich sich die Häufigkeit bewegungseinschränkender Maßnahmen deutlich unterschied. Andere Studien kommen ebenfalls zu dem Ergebnis, dass der Verzicht auf bewegungseinschränkende Maßnahmen nicht mit einem spürbar erhöhten Sturzrisiko einhergeht [6, 19] und somit die Anwendung bewegungseinschränkender Maßnahmen zur Sturzprophylaxe nicht notwendig ist.

Ein eindeutiger Prädiktor für den Einsatz sowohl sedierender Medikamente als auch bewegungseinschränkender Maßnahmen war die Versorgungsart. So war in der Kontrollgruppe die Wahrscheinlichkeit zu sedieren, etwa doppelt so hoch im Vergleich zur Station DAVID. Die Wahrscheinlichkeit für bewegungseinschränkende Maßnahmen war etwa 3‑mal höher. Dass der Einsatz bewegungseinschränkender Maßnahmen auf Stationen mit besonderen Versorgungskonzepten für PmD seltener vorkommt, konnte auch in anderen Untersuchungen gezeigt werden [30]. Gründe für die Unterschiede sind die verschiedenen Bausteine dieser Versorgungskonzepte [2]. Oft sind besondere Schulungsangebote ein wesentlicher Bestandteil von ihnen. Der geschulte Umgang mit PmD trägt dazu bei, dass das Pflegepersonal proaktiv auf die Patienten zugehen kann, noch bevor größere Unruhe oder Verhaltensauffälligkeiten entstehen. Studien zeigen die positiven Effekte von Schulungsmaßnahmen und verbesserter Versorgungs- und Lebensqualität bei PmD [14]. Obwohl der exakte Einfluss eines besonderen Versorgungskonzepts schwer zu quantifizieren ist, erscheint das Ergebnis durchaus plausibel. Ein erhöhter Personalschlüssel, besondere bauliche Begebenheiten oder auch spezielle Schulungsmaßnahmen sind essenzielle Maßnahmen zur Steigerung der Versorgungs- und Lebensqualität von PmD [12]. Nicht zuletzt ist die Lebensqualität von PmD deutlich höher, wenn auf Sedierung und bewegungseinschränkende Maßnahmen verzichtet wird [8].

Um ein solches Konzept umzusetzen, sind zusätzliche Zeit- und personelle Ressourcen nötig, die nicht immer gegenfinanziert werden [11]. Im Rahmen eines besonderen Angebots wie der Station DAVID fallen beispielsweise höhere Personal- als auch Behandlungskosten an. Gesundheitspolitische Maßnahmen sind notwendig, um für Krankenhäuser nicht nur Anreize, sondern realistische Möglichkeiten zu schaffen, eine Lösung für die stetig bedeutsamer werdende Patientengruppe „Menschen mit Nebendiagnose Demenz“ zu finden und entsprechende Lösungsansätze umzusetzen. Bei Demenzpatienten ist die Gefahr von Folgekomplikationen nachweislich erhöht; zudem sind Menschen mit Demenz häufiger im Krankenhaus als gleichaltrige Menschen ohne kognitive Einschränkungen [1, 17]. Daher ist eine verbesserte Versorgungs- und Lebensqualität von PmD im Krankenhäusern mit Nachdruck zu empfehlen. Die finanziellen Einsparungen durch Vermeidung von Folgekosten können die zusätzlichen Kosten, die durch erhöhten Personalaufwand im Krankenhaus entstehen, wahrscheinlich kompensieren.

### Limitationen

Die Ergebnisse haben einige Limitationen. Zum einen unterschieden sich die Patienten beider Krankenhäuser in verschiedenen Merkmalen, insbesondere hinsichtlich ihrer Mobilität. Um eine bestmögliche Vergleichbarkeit herzustellen, wurden die Modelle für diese und weitere Merkmale wie CCI adjustiert. Eine Ungleichverteilung von morbiden Patienten könnte eine weitere Einschränkung bezüglich der Vergleichbarkeit der Krankenhäuser darstellen. Jedoch zeigte der Vergleich von Hauptdiagnosen, dass keine der beiden internistischen Abteilungen deutlich morbidere Patienten behandelte. Weitere Limitationen sind die strukturellen Unterschiede beider Krankenhäuser, v. a. hinsichtlich des Personalschlüssels. Dies dürfte ein wesentlicher Grund für die festgestellten Unterschiede sein. Jedoch kann dies weniger als Verzerrung der Ergebnisse gesehen werden. Bei dem erhöhten Personalschlüssel handelt es sich um einen von mehreren Bausteinen des Versorgungskonzepts. Somit ist dies nicht per se als „struktureller Unterschied“ zu bewerten. Der Vergleich eines umfassenden Versorgungskonzepts für PmD zur Regelversorgung kann daher als „unfair“ wahrgenommen werden, obwohl es Teil der gesamten Intervention ist. Dennoch sollten Studien künftig weitere Kontrollgruppen oder eine Interventionsgruppe mit ähnlichen strukturellen Merkmalen einbeziehen, um mögliche Ergebnisverzerrungen durch Krankenhausunterschiede zu minimieren.

## Fazit für die Praxis

- Herausfordernde Verhaltensweisen sowie eingeschränkte funktionelle Kompetenz bei Patienten mit Demenz sind zentrale Gründe für den Einsatz sedierender Medikamente oder bewegungseinschränkender Maßnahmen in Akutkrankenhäusern.
- Demenzsensible Krankenhausstationen stellen einen wesentlichen Aspekt zur Verringerung dieser Maßnahmen und damit zur Verbesserung der Versorgungs- und Lebensqualität bei Patienten mit Demenz dar.
- Um eine besondere Wirksamkeit zu erreichen, sollten Versorgungskonzepte für demenzsensible Krankenhausstationen nicht nur punktuelle Maßnahmen, sondern verschiedene Bausteine umfassen. Dazu zählen neben der baulichen Gestaltung und räumlichen Aspekten auch demenzspezifische Schulungsangebote und ein angemessener Personalschlüssel.
- Zu klären bleibt, wie mögliche Mehrkosten (Baumaßnahmen, Personal) kompensiert werden können. Zumindest in dieser Studie gab es keine Hinweise darauf, dass die Aufenthaltsdauer durch spezielle Versorgungskonzepte signifikant verringert werden könnte.

## Footnotes

1 Die hier vorgestellten Maßnahmen sind grundsätzlicher, dauerhafter Bestandteil des Versorgungskonzepts und nicht auf die Zeit der wissenschaftlichen Evaluation begrenzt.
