# Erratum zu: Einsatz von sedierenden Medikamenten und bewegungseinschränkenden Maßnahmen bei Patienten mit Demenz im Akutkrankenhaus

**DOI:** 10.1007/s00391-021-01938-z

**Published:** 2021-07-14

**Authors:** Daniel Lüdecke, Christopher Kofahl

**Affiliations:** Institut für Medizinische Soziologie, Universitätsklinikum Hamburg-Eppendorf, Martinistraße 52, 20246 Hamburg, Deutschland

**Erratum zu:**

**Z Gerontol Geriat 2020** **53:138–144**

10.1007/s00391-020-01697-3

Der Artikel „Einsatz von sedierenden Medikamenten und bewegungseinschränkenden Maßnahmen bei Patienten mit Demenz im Akutkrankenhaus“ von Daniel Lüdecke und Christopher Kofahl wurde ursprünglich „online first“ ohne „Open Access“ auf der Internetplattform des Verlags publiziert. Nach der Veröffentlichung in Bd. 53, Heft 2, S. 138–144 hatten sich die Autoren für eine „Open-Access“-Veröffentlichung entschieden. Das Urheberrecht des Artikels wurde deshalb in © Der/die Autor(en) 2020 geändert. Dieser Artikel ist jetzt unter der Creative Commons Namensnennung 4.0 International Lizenz veröffentlicht, welche die Nutzung, Vervielfältigung, Bearbeitung, Verbreitung und Wiedergabe in jeglichem Medium und Format erlaubt, sofern Sie den/die ursprünglichen Autor(en) und die Quelle ordnungsgemäß nennen, einen Link zur Creative Commons Lizenz beifügen und angeben, ob Änderungen vorgenommen wurden. Die in diesem Artikel enthaltenen Bilder und sonstiges Drittmaterial unterliegen ebenfalls der genannten Creative Commons Lizenz, sofern sich aus der Abbildungslegende nichts anderes ergibt. Sofern das betreffende Material nicht unter der genannten Creative Commons Lizenz steht und die betreffende Handlung nicht nach gesetzlichen Vorschriften erlaubt ist, ist für die oben aufgeführten Weiterverwendungen des Materials die Einwilligung des jeweiligen Rechteinhabers einzuholen. Weitere Details zur Lizenz entnehmen Sie bitte der Lizenzinformation auf http://creativecommons.org/licenses/by/4.0/deed.de.

